# Global Offshore Wind Turbine Mapping in 2025 Using the CPEF Framework and Sentinel-1 SAR

**DOI:** 10.3390/s26154953

**Published:** 2026-08-05

**Authors:** Wenhe Liang, Yukan Jin, Boyu Liu, Tingting He

**Affiliations:** 1 College of Electronic Engineering, National University of Defense Technology, Hefei 230037, China; liangwenhegfkd@nudt.edu.cn (W.L.); 51253902026@stu.ecnu.edu.cn (Y.J.); lby@nudt.edu.cn (B.L.); 2 State Key Laboratory of Pulsed Power Laser Technology, Hefei 230000, China; 3 Advanced Laser Technology Laboratory of Anhui Province, Hefei 230000, China; 4 Department of Land Management, Zhejiang University, Hangzhou 310058, China

**Keywords:** offshore wind turbines, Sentinel-1 SAR, TM-CFAR, offshore wind energy, PCA-aligned peak profile fusion

## Abstract

**Highlights:**

**What are the main findings?**

- A CPEF framework was developed to map offshore wind turbines from global Sentinel-1 SAR imagery by combining TM-CFAR candidate extraction, EfficientNet-B0 refinement, and PCA-aligned peak profile fusion.
- The framework identified 16,270 offshore wind turbines worldwide in 2025, achieving an overall accuracy of 96.33% and an F1-score of 97.25%.

**What are the implications of the main findings?**

- The updated dataset improves the completeness of global offshore wind turbine mapping and supplements missing detections in rapidly developing regions like coastal China and the North Sea.
- The proposed framework provides a scalable and structurally interpretable SAR-based method for offshore infrastructure monitoring, marine spatial planning, and offshore wind resource assessment.

**Abstract:**

Accurate and up-to-date spatial information on offshore wind turbines (OWTs) is essential for offshore resource assessment, marine spatial planning, and environmental impact analysis. However, few existing datasets offer open access, global consistency, full-year 2025 coverage, and individual turbine-level spatial detail simultaneously. To address these gaps, we propose CPEF, a two-stage framework guided by constant false alarm rate (CFAR) detection that combines PCA-aligned peak profile features with EfficientNet-B0 image features for large-scale OWT detection using Sentinel-1 SAR imagery. We first composited multitemporal SAR observations into annual images to enhance persistent strong scatterers and suppress transient targets. A trimmed mean CFAR (TM-CFAR) was then used to generate high-recall candidate regions, followed by an EfficientNet-B0-based refinement model for candidate-level discrimination. To distinguish true turbines from false targets, PCA-aligned peak profile features were extracted along the principal and orthogonal directions and fused with deep visual features. CPEF detected 16,270 OWTs globally in 2025, with an overall accuracy of 96.33% and an F1-score of 97.25%. Compared with DeepOWT, our dataset identified additional turbines in regions such as Jiangsu, Shanghai, and the Dogger Bank area. Spatial analysis reveals that global OWTs primarily concentrate in nearshore Europe and coastal East Asia. In Asia, most turbines are in waters shallower than 20 m, whereas Europe has higher proportions in the 20–30 m and deeper depth ranges. These results demonstrate that CPEF offers an efficient, interpretable approach for global OWT mapping and provides an updated turbine-level dataset for spatial pattern analysis and offshore wind resource assessment.

## 1. Introduction

Global energy demand continues to rise with economic growth and industrialization. Yet the excessive use of traditional fossil energy has caused irreversible and severe pollution to the global ecological environment, with more than 75% of global greenhouse gas emissions originating from the consumption of fossil fuels [1,2,3]. To meet sustainability and climate goals, renewable energy has attracted great attention from governments and international organizations worldwide. Among various renewable energy sources, offshore wind energy has gradually become an important direction due to its advantages of stability, abundant resources, and cleanliness [4]. For instance, Germany has set an ambitious target of reaching an installed capacity of 30 GW by 2030 and 70 GW by 2045 [5], while the U.S. Department of Energy released the Offshore Wind Energy Strategy, which plans to achieve an installed scale of 110 GW by 2050 [6]. The rapid expansion of offshore wind energy has therefore drove a growing demand for accurate, up-to-date, and spatially explicit OWT datasets to support infrastructure monitoring, marine spatial planning, environmental assessment, and offshore wind resource evaluation [7].

Recent advances in remote sensing have provided reliable data sources for global OWT identification and dataset construction [8]. Among these, Sentinel-1 SAR imagery is particularly suitable for large-scale OWT detection due to its day-and-night, all-weather observations; wide spatial coverage; and open accessibility. Moreover, the metal structures of OWTs typically produce distinct strong backscattering responses in SAR images [9]. Yet global-scale OWT identification still faces interference from heterogeneous sea clutter, transient strong scatterers such as ships, and turbine-like false targets such as platforms and substations [10]. Traditional statistical detection methods offer high efficiency but have limited semantic discrimination. Deep learning methods demonstrate strong discrimination [11,12,13,14] but incur high computational costs when directly applied to large-scale images and underutilize the structural scattering characteristics of individual wind turbines. Currently, mainstream detection methods adopt a two-stage detection framework, yet they still struggle to balance the contradiction between processing efficiency in large scenarios and fine discrimination accuracy [15]. Therefore, to address the current limitation of global OWT data, there remains an urgent need to construct an automatic detection framework that balances large-scale search efficiency, the ability to suppress complex turbine-like false targets, and structural interpretability.

Existing offshore wind turbine (OWT) datasets and databases provide valuable information for offshore wind energy studies, but they differ substantially in data sources, spatial units and intended applications. The Global Wind and Solar Farms Dataset [16] offers an open global inventory of wind and solar installations derived from OpenStreetMap data, providing farm-level spatial information and auxiliary attributes such as estimated capacity. However, its completeness may vary across regions, as it relies on volunteered geographic information rather than direct satellite image interpretation. UKREPD [17] records project-level information for renewable energy developments, including development status, installed capacity, turbine number, and planning records. EMODnet [18] provides harmonized offshore wind farm (OWF) information for European seas, including wind farm locations or boundaries, operational status, turbine number, power, and distance to coast. Although these open-source datasets are useful for project statistics, infrastructure inventories and marine spatial planning, their direct applicability to the development and evaluation of image-based individual OWT detection algorithms is limited, because of the general lack of image-level samples, turbine-scale target features and false alarm annotations. Meanwhile, commercial databases, such as The Wind Power Database [19] and 4C Offshore Database [20], contain more systematic industry information, but their restricted accessibility limits reproducible use in open research.

In addition, remote-sensing-based detection provides a more scalable solution for large-area OWT mapping. For example, the global OWT dataset derived from Sentinel-1 SAR time-series imagery uses yearly SAR compositing and an adaptive thresholding strategy to extract turbine locations [21], which is efficient and interpretable, and it benefits from the all-weather, day-and-night imaging capability of SAR data. Nevertheless, threshold-based SAR mapping remains sensitive to sea clutter, imaging geometry, nearshore background complexity and turbine-like bright targets. To address these limitations, deep learning methods have been introduced to significantly improve feature representation compared with purely threshold-based approaches, such as DeepOWT [22], a method that employs a two-stage cascaded convolutional neural network to detect offshore wind turbine infrastructure from Sentinel-1 imagery. However, deep-learning-based approaches generally suffer from reduced generalization when applied across different coastal environments, turbine layouts, and SAR acquisition conditions. Above all, current research lacks a robust and scalable SAR-based detection framework that can maintain high recall in large-area candidate generation while suppressing turbine-like false alarms.

To address the above limitations, we developed a CPEF framework based on Sentinel-1 SAR imagery. Our main contributions are as follows. (1) We proposed CPEF, a two-stage SAR-based OWT mapping framework that integrates TM-CFAR candidate generation, EfficientNet-B0 refinement and PCA-aligned peak profile feature fusion. The framework transforms global-scale OWT mapping from full-scene dense inference to candidate-level discrimination, thereby reducing redundant background processing while maintaining sensitivity to potential turbine targets. (2) PCA-aligned peak profile features were introduced to explicitly characterize the local axial scattering structure of individual OWT candidates in Sentinel-1 SAR imagery. By fusing these interpretable structural descriptors with deep visual features, we enhanced the discrimination between true OWTs and strong-scattering turbine-like false targets, such as offshore platforms and substations. (3) An updated global turbine-level OWT inventory for 2025 was produced, and the spatial distribution and bathymetric characteristics of global offshore wind development were analyzed. The resulting dataset provides an open and reproducible data basis for offshore infrastructure monitoring, marine spatial planning, and resource assessment. The remainder of this paper is organized as follows: Section 2 presents the data and methodology, Section 3 reports the global mapping and validation results, Section 4 discusses the findings, and Section 5 concludes.

## 2. Materials and Methodology

### 2.1. Overview of Methodology

This study proposes CPEF, an OWT detection framework that combines efficient large-scene detection with fine-grained target discrimination. In the first stage, a conventional statistical detection method rapidly localizes potential turbine regions in Sentinel-1 SAR imagery. Specifically, an annual SAR composite for OWT detection in 2025 is first constructed. A Refined Lee filter is then applied to suppress speckle noise while preserving the strong-scattering structures of OWTs. The filtered images are subsequently fed into a trimmed mean constant false alarm rate (TM-CFAR) detector, which generates a binary mask of candidate regions. Connected component analysis is then performed on the binary mask to standardize candidate region representation and reduce interference from isolated anomalously bright pixels. Components smaller than 300 pixels are removed, yielding OWT candidate regions with high recall.

Using the generated candidate regions as input, the second stage introduces a deep-learning-based refinement strategy to improve the discrimination accuracy between real turbines and turbine-like false targets. Unlike methods that rely solely on local image texture for candidate classification, the second stage further explicitly models the structural scattering signatures of candidate targets. For each candidate mask, the largest connected component is retained to reduce the influence of fragmented noise on subsequent shape analysis. PCA is then utilized to estimate the orientation of the candidate region’s principal axis. Pose normalization subsequently transforms candidates with different orientations into a unified reference coordinate system for comparison. Based on the aligned candidates, peak profile features along the principal axis and its perpendicular direction are extracted to characterize the spatial organization of scattering peaks These features provide explicit structural priors that a purely image-based branch may not capture reliably.

**Figure 1:**
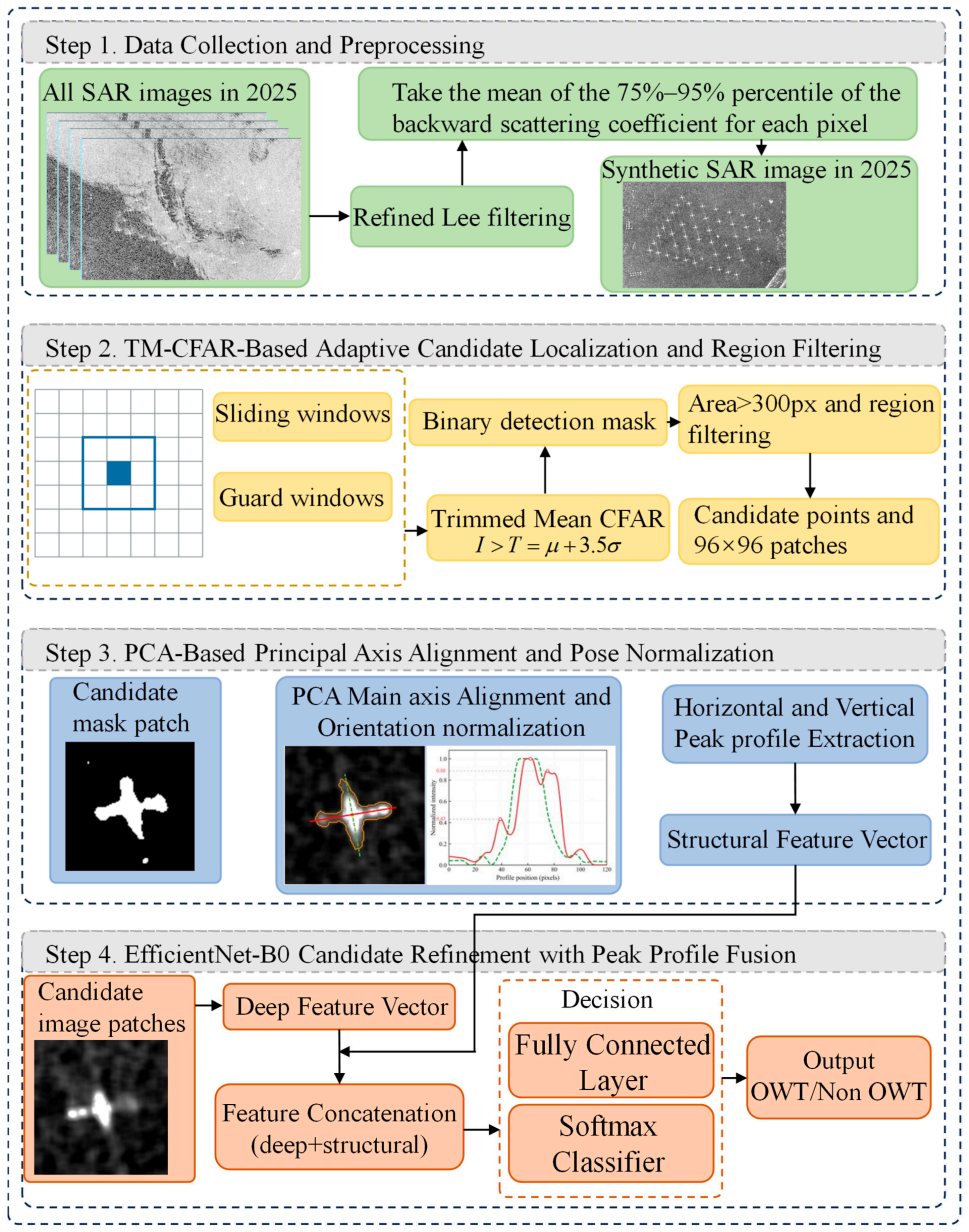
Architecture of CPEF framework.

Finally, the cropped candidate image patches are fed into an EfficientNet-B0 classification network and fused with the PCA-aligned peak profile features for discrimination, achieving the precise identification of true OWT regions. As illustrated in Figure 1, the overall framework comprises an image preprocessing module and a two-stage detection pipeline. It consists of four sequential steps: (1) Data Collection and Preprocessing; (2) TM-CFAR-Based Adaptive Candidate Localization and Region Filtering; (3) PCA-Based Principal Axis Alignment and Peak Profile Discrimination; and (4) EfficientNet-B0 Candidate Refinement with Peak Profile Fusion.

### 2.2. Data Collection and Preprocessing

Sentinel-1 GRD SAR images between 1 January and 31 December 2025 were obtained from the Google Earth Engine COPERNICUS/S1_GRD image collection. This study used IW-mode Sentinel-1 GRD products and selected the VV co-polarized sigma-nought (σ^0^) backscatter coefficient for OWT detection. Given that OWTs are mainly concentrated in nearshore shallow waters, the imagery was clipped to the maritime zones of coastal states, including internal waters, territorial seas, contiguous zones, and exclusive economic zones (EEZs). EEZ boundary vector data were obtained from the Flanders Marine Institute database.

Speckle noise, inherent to the SAR coherent imaging mechanism, causes strong random fluctuations in the sea surface background of raw images and degrades the reliability of subsequent pixel-wise temporal compositing and candidate extraction. Therefore, a Refined Lee filter was applied to the annual SAR data. The filter adaptively suppresses speckle noise based on local statistical properties, smoothing homogeneous sea surface areas while preserving the edges and local structures of strong scatterers such as wind turbines. The Refined Lee filter used a 7 × 7-pixel window, consistent with recent SAR despeckling and Sentinel-1 application studies. These studies reported or adopted a 7 × 7 window as an effective compromise between speckle suppression and spatial detail preservation [23,24,25].

**Figure 2:**
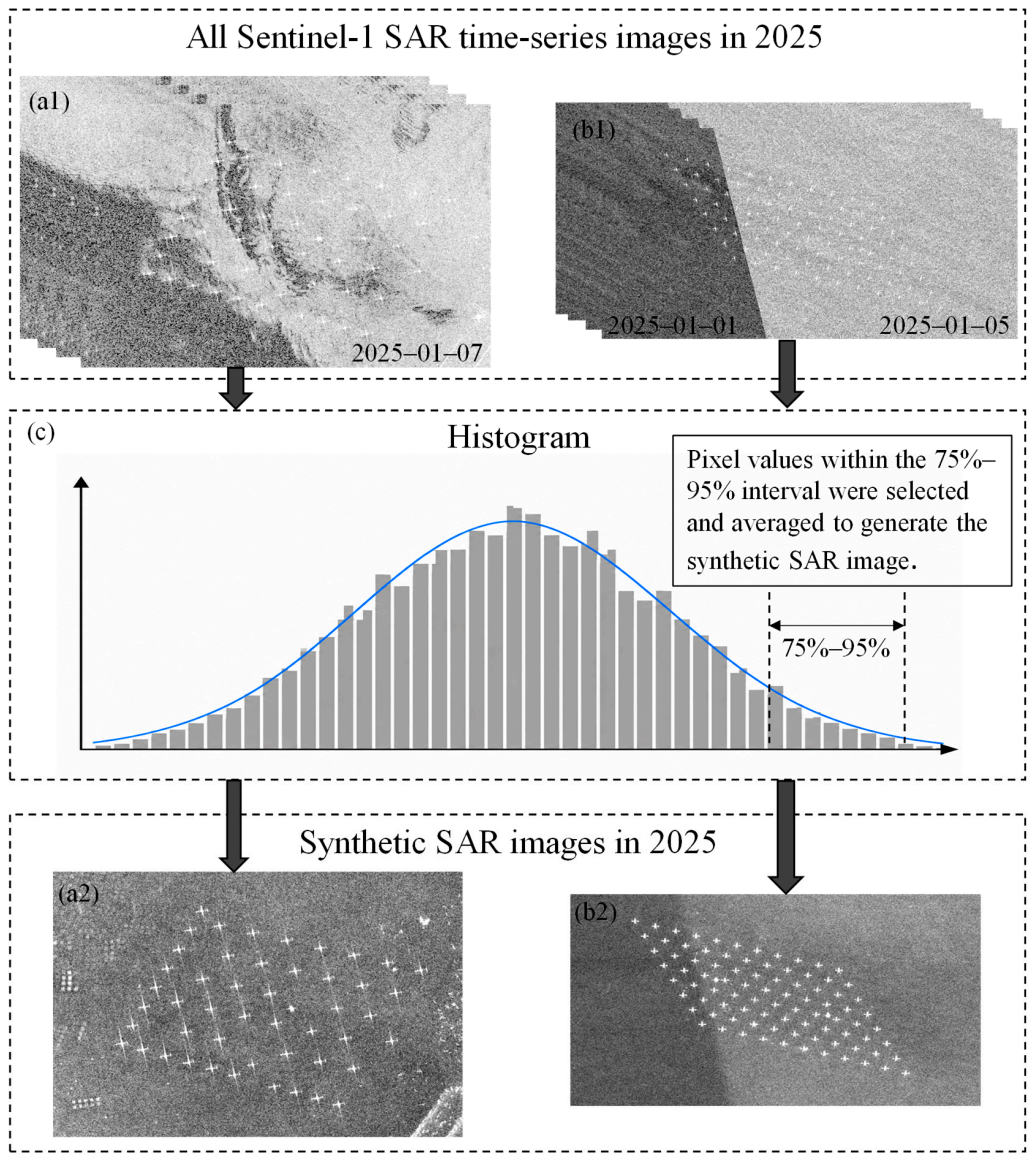
Pixel-based annual SAR quantile compositing. (**a1**,**b1**) show representative Sentinel-1 SAR observations acquired in 2025; (**c**) illustrates the pixel-wise backscatter distribution and the averaging of values within the 75th–95th percentile interval; and (**a2**,**b2**) present the resulting annual SAR composite images.

After Refined Lee filtering, a pixel-based quantile compositing method was applied to generate the annual SAR composite. Specifically, all valid Sentinel-1 observations acquired throughout 2025 were collected for each pixel location to construct an annual backscatter time series. Observations within the 75th–95th percentile interval of each time series were then selected, and their mean value was assigned to the corresponding pixel in the composite image. This strategy uses all valid annual observations at each pixel location, thereby enhancing stable strong scatterers such as OWTs while suppressing dynamic strong-scattering interference. Averaging the observations within the 75th–95th percentile interval limits the influence of low-percentile values caused by sea-state variability and residual noise. It also reduces contamination from extremely high-percentile values associated with transient targets such as ships [26]. Compared with the original SAR observations in Figure 2(a1,b1), the annual composites in Figure 2(a2,b2), generated using the strategy shown in Figure 2c, suppress sea surface fluctuations and transient ship returns, making OWT signatures more stable and distinguishable. All valid observations were first co-registered to a common spatial grid and then composited to generate a seamless annual SAR composite. The proposed annual compositing strategy is not restricted to 2025. For other years, the same procedure can be applied by replacing the input Sentinel-1 image collection with observations from the corresponding year, provided that sufficient valid SAR acquisitions are available. Accordingly, both the annual compositing strategy and the overall CPEF framework are temporally transferable and applicable to multi-year OWT mapping. Specifically, all preprocessing steps described above were implemented on the Google Earth Engine platform using JavaScript.

### 2.3. TM-CFAR-Based Adaptive Candidate Localization and Region Filtering

Following speckle noise suppression, a TM-CFAR detector was employed to generate candidate OWT regions. In contrast to fixed global thresholding, TM-CFAR adaptively estimates a detection threshold based on the local statistical properties of the pixel neighborhood. It is therefore better suited to SAR scenes with spatially heterogeneous sea surface scattering and pronounced local background fluctuations. For each pixel under test, an outer training window and an inner guard window were defined. Based on the observed spatial characteristics of representative OWT scattering responses in Sentinel-1 imagery, we set the guard window side length to 41 pixels. This size effectively covers the main bright response of an individual OWT and its immediately affected strong-scattering neighborhood, thereby preventing target energy from contaminating the background statistics. The outer training window side length was set to 121 pixels. The resulting training ring provided sufficient surrounding sea surface samples for stable local clutter estimation while avoiding the excessive inclusion of nonlocal background variations. The guard region isolates the target body and its immediate strong response. The training ring, obtained by subtracting the guard region from the outer window, was then used to estimate the local background characteristics.

Because OWTs are often arranged regularly and densely within an OWF, traditional mean CFAR training rings might include neighboring turbines or other bright scatterers, thereby inflating the background estimate. To improve candidate generation robustness in dense turbine scenes, a trimmed mean strategy was used to estimate the training ring statistics. The pixels within the training ring were first sorted by intensity, after which the brightest 20% were discarded. The 20% trimming proportion is a commonly used setting in robust trimmed mean estimation for reducing the influence of extreme values and outliers [27]. Then the background mean and standard deviation were computed from the remaining pixels to construct an adaptive detection threshold

(1) $$T=\mu_{\mathrm{trim}}+k\sigma_{\mathrm{trim}}$$

where $\mu_{\mathrm{trim}}$ and $\sigma_{\mathrm{trim}}$ denote the trimmed mean and standard deviation, respectively, and k is a threshold coefficient, here set to 3.5. This value was adopted as a conservative anomaly detection threshold, following the commonly used 3.5 criterion in robust Z-score-based outlier detection [28]. Previous wind turbine remote sensing studies have also used a similar threshold to identify potential turbine pixels while reducing false positives [8]. A pixel was classified as a potential target response when its intensity exceeded this threshold. By removing high-intensity outliers from the training ring, this strategy effectively reduces the influence of nearby turbines, isolated bright spots, and other strong scatterers on background statistical estimation, thereby enhancing the recall and stability of the candidate generation stage.

TM-CFAR outputs a pixel-level binary detection mask. To convert the discrete detection responses into region-level candidates suitable for subsequent classification, connected component analysis was performed on the binary mask. Using an 8-connectivity rule, spatially adjacent target response pixels were grouped into separate candidate regions. Then geometric attributes—including area, bounding box width, bounding box height, aspect ratio, and centroid position—were computed for each region. This process converts the pixel-wise detection results into a unified region-level representation, which supports subsequent image patch cropping and classification.

**Figure 3:**
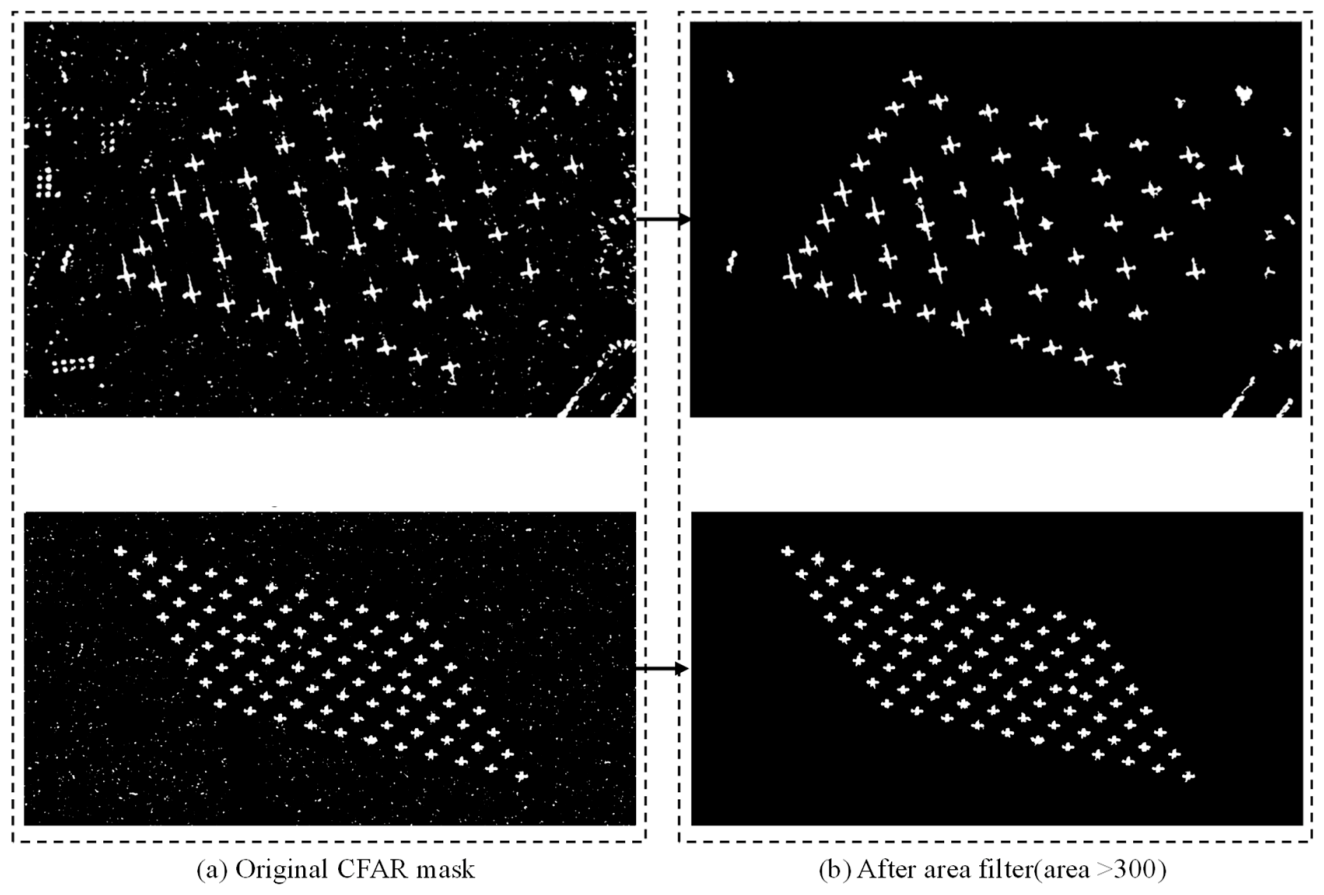
Candidate region generation and area filtering using TM-CFAR. (**a**) shows original TM-CFAR binary masks containing OWT responses and scattered small-scale false responses, while (**b**) shows the filtered masks after the removal of connected components with areas not exceeding 300 pixels.

Because the CFAR detection results may still contain small-scale false responses caused by residual sea clutter, isolated bright spots, or local anomalous scattering, we applied an area constraint after connected component extraction. Specifically, only candidate connected components with an area greater than 300 pixels were retained, while smaller isolated noise and fragmented responses were discarded. This filtering preserved high target recall while substantially reducing noise interference and the number of redundant candidates. It consequently provided more target-relevant input regions for the fine discrimination stage. Figure 3 illustrates the candidate region generation and filtering results of the TM-CFAR stage. As shown in Figure 3a, the adaptive detector extracts prominent scattering responses around OWTs. Based on 8-connected-component analysis and area-based filtering, Figure 3b shows that scattered noise is effectively removed, while the main OWT candidates are retained.

### 2.4. PCA-Based Principal Axis Alignment and Peak Profile Encoding

#### 2.4.1. Largest Component Purification for Structural Stability

After candidate regions were generated, the local binary mask corresponding to each candidate was refined by retaining only the largest connected component, thereby enhancing the stability of the subsequent geometric analysis. Although the preceding stage removed most small-scale noise candidates, the retained masks could still contain internal fragments, weakly connected branches, or boundary-adherent components. These spurious responses, while not decisive for candidate retention, could alter the spatial distribution of foreground pixels and consequently degrade the reliability of principal axis orientation estimation.

To address this issue, connected component analysis was reapplied to each candidate mask, and only the connected component with the maximum area was preserved as the purified main body. Formally, given K connected components $\left\{C_{1\;},C_{2}\;,\ldots ,C_{K}\;\right\}$ within a candidate mask, the retained main body region is defined as follows:

(2) $$C^{*}=\arg\;\max_{C_{k}}|C_{k}|$$

where $C_{k}$ denotes the area of the k-th component. After this refinement, PCA-based principal axis estimation operates only on the dominant structure rather than on a mixture of the main body and discrete spurious responses. This provides a more robust structural basis for subsequent orientation normalization and peak profile feature extraction.

#### 2.4.2. PCA-Based Principal Axis Estimation and Pose Normalization

After the largest connected component refinement was completed, PCA was further employed to estimate the principal axis orientation of each candidate region and to perform pose normalization accordingly. This step is critical for constructing structural priors; its purpose is not merely to obtain a geometric orientation angle but rather to establish a unified directional reference coordinate system for the subsequent extraction of peak profile features. It should be noted that PCA was not used for dimensionality reduction in this study. Instead, eigenvalue decomposition was performed on the two-dimensional coordinate covariance matrix of foreground pixels, and only the first principal component, corresponding to the largest eigenvalue, was used to estimate the principal axis orientation. The orthogonal direction was then defined as perpendicular to the first principal component. Candidate regions generated by TM-CFAR typically have different orientations in the original SAR imagery. Consequently, the local scattering structures of the same target class may appear under different rotational poses. If transverse or longitudinal profiles were extracted directly in the original image coordinate system, the peak structures across different candidates would lack consistent geometric semantics, consequently rendering the profile statistics incomparable and weakening their discriminative power. We therefore introduced a PCA-based principal axis estimation and orientation normalization strategy. This strategy maps candidates with different orientations into a unified pose reference frame, so subsequent profile encoding is not affected by their original orientations.

PCA-based principal axis estimation relies on the spatial distribution of the foreground pixels in the purified main body mask. Let the set of foreground pixel coordinates in a candidate region be ${\left\{(x_{n},y_{n})\right\}}_{n=1}^{N}$ and its centroid be $\left(\bar{x},\bar{y}\right)$. To avoid unstable covariance estimates for extremely small connected components, direction estimation was skipped when the number of valid foreground pixels N was less than 30, and the rotation angle was assigned a default value. When N ≥ 30, the centered coordinates and the corresponding two-dimensional covariance matrix were computed as follows:

(3) $${\tilde{p}}_{n}=\left[\begin{array}{c} x_{n}-\bar{x}\\ y_{n}-\bar{y} \end{array}\right],\;C=\frac{1}{N-1}\sum_{n=1}^{N}{\tilde{p}}_{n}{\tilde{p}}_{n}^{T}$$

Let $v_{1}={\left[v_{x},v_{y}\right]}^{T}$ denote the unit eigenvector of C corresponding to the largest eigenvalue. The raw principal axis angle and the pose normalization rotation angle were then defined as

(4) $$\phi =\mathrm{atan}2(v_{y},v_{x}),\;\theta =90^{{}^{\circ}}-\phi$$

where θ was constrained to the range $\left[-90^{{}^{\circ}},90^{{}^{\circ}}\right]$ to eliminate periodic ambiguity in the angular representation. Candidate regions with different original orientations were thereby mapped into a unified directional reference frame. Profiles extracted along and orthogonal to the principal axis consequently had consistent structural meanings across candidates.

Based on the resulting normalized rotation angle, a rigid rotation was applied to the local grayscale image and the binary mask using the candidate image patch center as the rotation center. This process yielded a direction-normalized candidate representation. During rotation, bilinear interpolation was employed for the grayscale image to preserve the continuity of profile intensity variations. For the binary mask, nearest-neighbor interpolation was adopted to prevent the connected structure from being degraded by smooth interpolation. Direction normalization removes orientation differences among candidate regions and provides a unified, stable, and geometrically comparable coordinate basis for constructing peak profile features.

### 2.5. EfficientNet-B0 Candidate Refinement with Peak Profile Fusion

**Figure 4:**
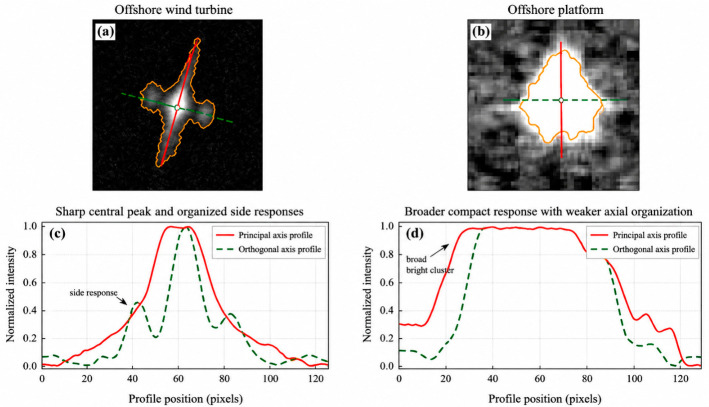
A comparison of local SAR responses between an OWT and an offshore platform. (**a**,**b**) show the local SAR responses of the OWT and offshore platform. (**c**,**d**) present the corresponding normalized intensity profiles along the two axes. The OWT exhibits a sharp central peak and organized side responses, whereas the offshore platform presents a broader compact response with weaker axial organization.

Following TM-CFAR candidate generation, the full-scene SAR image was reduced to a limited set of high-response candidate regions. Nevertheless, this stage still relied on local statistical contrasts between targets and the sea surface background. Consequently, it could retain strongly scattering non-turbine installations, such as offshore substations and oil platforms. Compared with these interfering targets, OWTs typically exhibit a more clearly defined axial organization in their local SAR responses. Their main bodies generally appear as sharp central bright-scattering clusters accompanied by relatively concentrated extended scattering along the principal response direction. In contrast, platform-type targets tend to have a larger spatial scale and a broader, cluster-like strong-scattering response. Although both categories appear as high-backscatter targets, the OWT in Figure 4a,c exhibits a sharper peak and more distinct axial structure than the offshore platform in Figure 4b,d. Based on this difference, after PCA-based principal axis estimation and pose normalization, the central profiles along the principal and orthogonal axes were extracted in a unified coordinate system to construct a five-dimensional aligned peak profile feature vector:

(5) $$f_{\mathrm{prof}}={\left[f_{1},f_{2},f_{3},f_{4},f_{5}\right]}^{T}$$

where $f_{1}$ and $f_{2}$ denote the numbers of valid peaks in the transverse and longitudinal profiles, respectively, describing the response complexity along the two principal directions; $f_{3}$ represents the intensity ratio between the main peak and the side peaks in the transverse profile, capturing the prominence of the central response relative to the flanks; $f_{4}$ quantifies the symmetry between the left and right side peaks in the transverse profile, characterizing the structural balance orthogonal to the principal axis; and $f_{5}$ measures the average distance between the main peak and the side peaks, describing the spatial scale of the lateral response.

This feature formulation shifts candidate discrimination from asking whether the intensity is sufficiently high to determining whether the structural organization conforms to the characteristic local scattering pattern of an OWT. For oil platforms, substations, and other offshore infrastructure, even when their local backscatter intensity is comparably strong, their profile peak morphology, the degree of central broadening, and the ratio between transverse and longitudinal responses typically diverge from those of OWTs. Consequently, peak profile features provide explicit structural information that complements image texture during the subsequent fine classification stage. Candidate discrimination therefore no longer relies solely on implicit deep representations but simultaneously exploits a local scattering descriptor that carries geometric constraints and physically interpretable meaning.

Building on these structural priors, we constructed a deep-learning-based candidate refinement network that fuses them with deep visual features. Through hierarchical convolutional feature extraction, the model learns complex nonlinear representations within candidate regions and captures subtle differences between OWTs and challenging turbine-like false targets. Because the second stage operates on candidate image patches rather than full-scene SAR imagery, it is well suited to classification with a lightweight convolutional network, preserving discriminative power while controlling computational cost. Accordingly, EfficientNet-B0 was selected as the deep feature extraction backbone for the image branch. With a modest number of parameters, the network offers efficient representation learning, while its hierarchical convolutional architecture extracts local texture and multiscale scattering patterns from candidate regions [26].

In summary, a dual-branch discrimination network was constructed that fuses image representations with peak profile structural features. The image branch takes the candidate region grayscale image patch $x_{i}\in {\mathbb{R}}^{H\times W}$ as input. Considering that a SAR candidate patch is single-channel, it is first replicated into a three-channel form, uniformly resized to 224 × 224, and then fed into the backbone network for deep visual feature encoding. In parallel, the structural branch takes the peak profile features as input and characterizes the scattering organization along and orthogonal to the principal axis. For each candidate region, the image branch extracts a deep visual feature vector $z_{i}^{\mathrm{img}}$, whereas the structural branch provides a PCA-aligned peak profile feature vector $z_{i}^{\mathrm{feat}}$. The two feature vectors are concatenated to form the fused representation $z_{i}$, which is then passed through a classification head to output the probability that the candidate region belongs to an OWT:

(6) $$z_{i}=\left[z_{i}^{\mathrm{img}}∥z_{i}^{\mathrm{feat}}\right],p_{i}=\sigma \left(H_{\mathrm{cls}}(z_{i})\right)$$

where [⋅||⋅] denotes the feature concatenation operation, $H_{\mathrm{cls}}(\cdot )$ denotes the learnable classification head that maps the fused feature vector to a scalar logit, σ(⋅) denotes the sigmoid activation function that converts the logit into the predicted probability, and $p_{i}$ represents the predicted probability that the candidate region belongs to an OWT.

**Figure 5:**
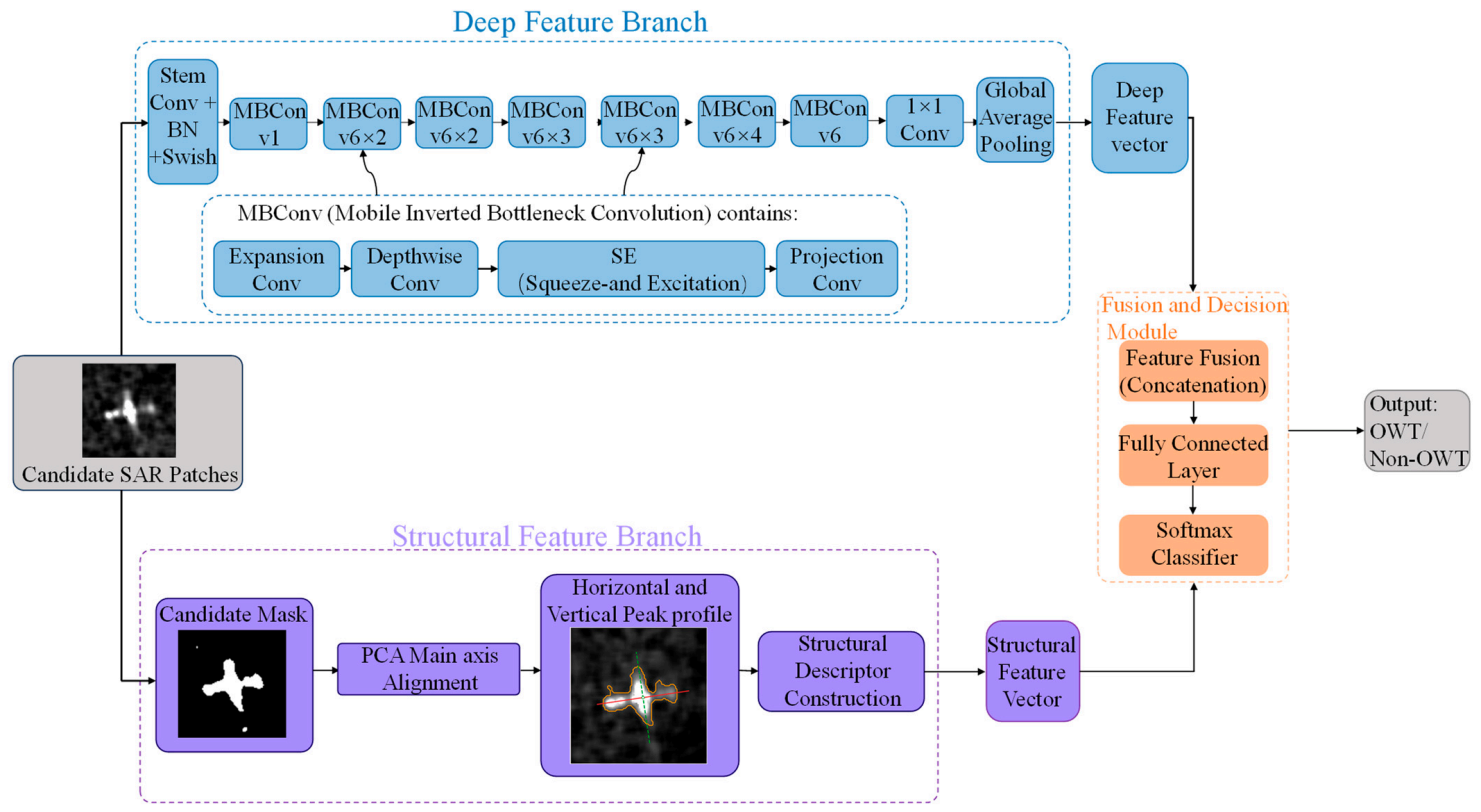
Architecture of EfficientNet-B0 fine classification network fusing image features with PCA-aligned peak profile features.

Through this design, candidate refinement no longer relies entirely on EfficientNet-B0 to learn discriminative cues implicitly from raw image patches. Instead, it explicitly incorporates peak shape information tied to the local structure of wind turbines. The image branch characterizes local texture, bright scatterer distribution, and surrounding contextual differences. The peak profile branch complements these features by encoding the relationships between the main response and its two flanks, the contrast between the main and side peaks, and the response organization along and orthogonal to the principal axis. The two branches complement each other at the feature level, enabling the model not only to recognize whether a candidate region exhibits a prominent bright scattering response but also to further judge whether this bright scattering conforms to the characteristic local structural pattern of an OWT. This fusion strategy thereby improves the stability of candidate refinement in the presence of complex backgrounds and strong-scattering turbine-like false targets. During training, the image branch was initialized with pretrained EfficientNet-B0 weights, and its final classification layer was replaced for the binary candidate refinement task. The fusion model was trained with the AdamW optimizer for 50 epochs, using an initial learning rate of 0.001, a weight decay of 5 × 10^−4^, and a batch size of 32. A cosine learning rate decay strategy with a short warm-up stage was adopted to stabilize early training, and binary cross-entropy loss was used as the optimization objective. Figure 5 illustrates the architecture of the fusion model.

## 3. Results

### 3.1. Spatial Distribution of OWTs

**Figure 6:**
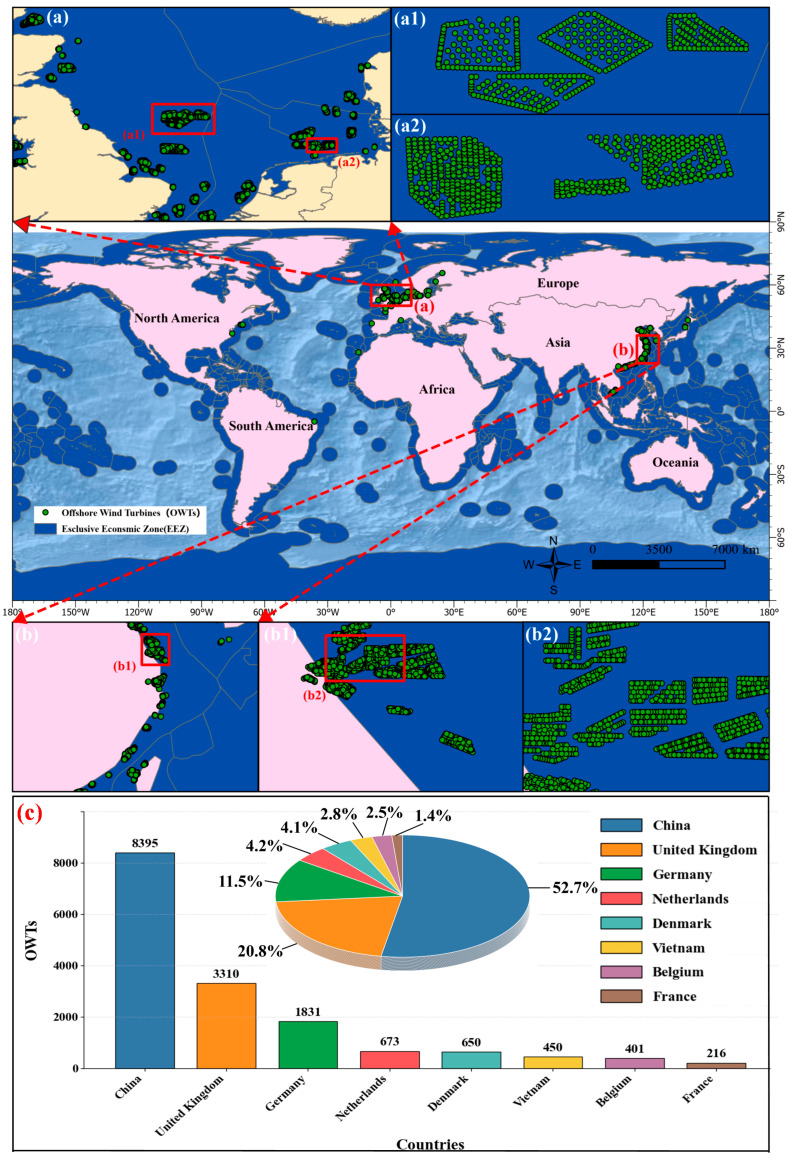
The spatial distribution of global OWT detections in 2025. The central map shows the global distribution of detected OWTs. (**a**,**b**) provide enlarged views of the North Sea and coastal China, respectively; (**a1**,**a2**,**b1**,**b2**) show representative OWT array layouts within these regions; and (**c**) presents the OWT counts and corresponding percentage shares among the eight countries with the highest numbers of detected OWTs.

Figure 6 presents the global spatial distribution of OWT detections in 2025. By the end of 2025, we identified a total of 16,270 OWTs. Compared with 2022, the global number of OWTs increased by 3858, corresponding to an average annual increase of approximately 1286 turbines over the three-year period. As shown in Figure 6a,b, OWTs are concentrated mainly in the North Sea and coastal China, where they typically form regular arrays. In contrast, relatively few OWTs are distributed off the coasts of the Americas, Africa, and Oceania.

Local zoomed-in views further show that most of the detected turbine points lie within nearshore EEZs and exhibit regular array-like layouts, including rectangular, parallelogram, trapezoidal, and row–column arrangements characteristic of typical OWFs. These regular distributions are closely associated with the joint consideration of wake effects, turbine spacing, maritime boundaries, and navigation safety during wind farm planning. These results indicate that our inventory captures both the large-scale spatial clustering of global OWTs and the structured layouts of individual wind farms.

National-level statistics reveal that global OWTs are highly concentrated in a small number of leading countries. China accounted for the largest share, with 8395 turbines, representing 51.6% of the global total. The United Kingdom and Germany followed with 3310 (20.3%) and 1831 (11.3%) turbines, respectively. The Netherlands and Denmark had 673 and 650 turbines, accounting for approximately 4.1% and 4.0%, respectively. The top three countries collectively accounted for 83.2% of all detections, while the top five reached 91.3%. These results demonstrate that the current global spatial distribution of OWTs is highly concentrated in a small number of countries with early and large-scale offshore wind development. This pattern is broadly consistent with the development pattern of the global offshore wind industry [21].

### 3.2. Bathymetric Distribution Patterns of OWTs

Water depth is a critical factor influencing the siting and spatial configuration of OWTs. Different depth intervals are associated with different foundation types, construction complexities, operation and maintenance costs, and levels of engineering suitability. Analyzing the water depth distribution of OWTs therefore helps reveal the spatial preferences and engineering constraints shaping offshore wind development across countries and regions.

**Figure 7:**
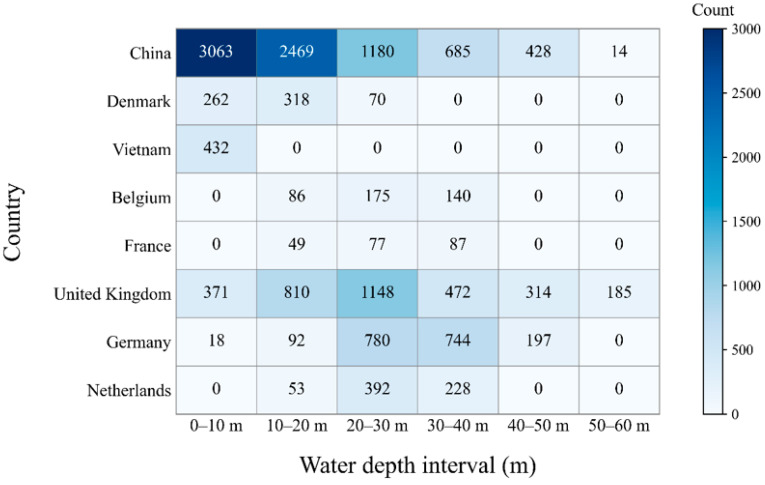
Water depth distribution of OWTs for major global offshore wind countries.

Figure 7 presents the distribution of OWTs across water depth intervals in the eight countries with the largest total turbine counts. Overall, Asian countries concentrate their OWTs predominantly in shallower waters, whereas those in European countries have a stronger presence in intermediate depth ranges. China, for instance, had 3063 turbines in the [0, 10 m] interval but only 14 turbines in the [50, 60 m] interval. This distribution indicates a pronounced decline in turbine count with increasing water depth. Vietnam’s OWTs were almost entirely located within the [0, 10 m] shallow interval, with virtually no turbines beyond 10 m. In contrast, OWTs in several major European offshore wind countries were concentrated mainly in the [20, 30 m] interval. The United Kingdom, Germany, Belgium, and the Netherlands recorded their highest turbine counts in this range, with 1148, 780, 175, and 392 turbines, respectively. Denmark’s OWTs were primarily concentrated in the [10, 20 m] interval, which contained 318 turbines. These results demonstrate clear inter-country differences in water depth distribution: Asian countries generally favor shallow nearshore development, while European nations have established mature, large-scale deployments in intermediate-depth waters.

### 3.3. Technical Validation

#### 3.3.1. Evaluation Metrics

To quantitatively evaluate the performance of the CPEF framework, four metrics were introduced: overall accuracy (OA), user’s accuracy (UA), producer’s accuracy (PA), and the F1-score (F1). OA represents the proportion of all samples that are correctly classified; UA measures the proportion of samples predicted as positive that are truly positive; PA indicates the proportion of true positive samples that are correctly detected; and F1, defined as the harmonic mean of UA and PA, provides a combined measure of the precision and completeness of the detection results. The corresponding formulas are as follows:

(7) $$OA=\frac{TP+TN}{TP+FP+FN+TN}$$

(8) $$UA=\frac{TP}{TP+FP}$$

(9) $$PA=\frac{TP}{TP+FN}$$

(10) $$F1=\frac{2\cdot UA\cdot PA}{UA+PA}$$

where TP (true positive) denotes the number of positive samples correctly predicted, TN (true negative) the number of negative samples correctly predicted, FP (false positive) the number of negative samples incorrectly predicted as positive, and FN (false negative) the number of positive samples incorrectly predicted as negative.

#### 3.3.2. Performance Assessment

An independent validation dataset was constructed to evaluate the accuracy of the 2025 OWT mapping results. To ensure spatial representativeness, samples were selected from coastal China and the North Sea, two major offshore wind regions. Reference labels were generated through manual visual interpretation by jointly examining multitemporal Sentinel-1 observations, high-resolution optical images, and existing OWT inventories as auxiliary references. In total, 300 validation samples were evaluated, including 200 OWT samples and 100 non-OWT samples. Based on the resulting confusion matrix, OA, UA, PA, and the F1-score were calculated to quantitatively assess detection accuracy.

The validation results demonstrate that the proposed two-stage detection framework performed well in identifying OWTs. Specifically, OA and the F1-score reached 96.33% and 97.25%, respectively, while PA and UA were 97.01% and 97.50%, respectively. These results indicate that the method not only maintains high detection capability but also effectively suppresses false positives, thereby achieving high precision and stable detection performance.

**Figure 8:**
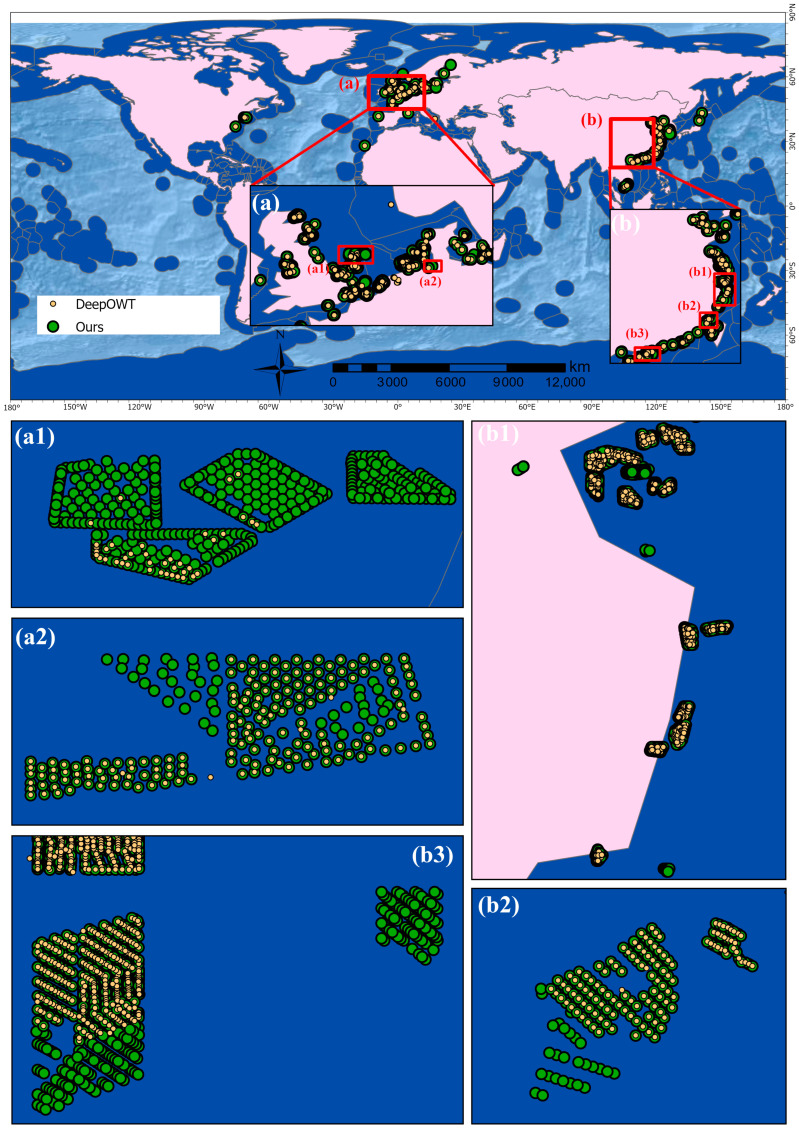
Spatial comparison between our detection results and the DeepOWT dataset. The main panel shows the global comparison; (**a**) enlarged comparison in the North Sea region; (**b**) enlarged comparison along coastal China; (**a1**,**a2**,**b1**–**b3**) representative local comparisons highlighting OWTs additionally identified by the proposed method. DeepOWT turbines are represented by yellow-filled circles, whereas the proposed detections are represented by green-filled circles.

To further evaluate the completeness of the detection results, we compared the derived OWT point locations with the deep-learning-derived OWT (DeepOWT) dataset. DeepOWT is a global OWT inventory produced from Sentinel-1 SAR imagery using deep learning methods. Its latest version provides the geographic locations of global OWTs through 2025, making it, to the best of our knowledge, the only publicly available and up-to-date global OWT dataset. Figure 8 compares our results with the DeepOWT dataset at the global and regional scales. DeepOWT turbines are shown as yellow-filled circles, whereas our detections are shown as green-filled circles. The enlarged panels in Figure 8a,b, together with the local comparisons in Figure 8(a1,a2,b1–b3), show that our method identifies additional OWTs not detected by DeepOWT. DeepOWT identified 15,606 turbines, whereas our method detected 16,270 OWTs. This indicates improved data completeness and spatial coverage in our results. Local comparisons show that DeepOWT exhibits omissions in regions such as Jiangsu and Shanghai in China and the Dogger Bank area in the North Sea, whereas our results are able to supplement OWT point locations in these areas. This comparison thus indicates that the 2025 mapping result can supplement existing global OWT inventories and provide an updated annual inventory for the target year.

## 4. Discussion

### 4.1. Implications of 2025 Global OWT Mapping Results

We identified a total of 16,270 OWTs worldwide, and the comparison with DeepOWT showed that the proposed dataset supplements missing turbine detections in regions such as Jiangsu, Shanghai, and the Dogger Bank area. These additional detections indicate that the CPEF framework can improve the completeness of global OWT mapping, particularly in rapidly developing coastal regions and densely clustered wind farms.

The spatial distribution results further reveal that global OWT deployment remains highly uneven. OWTs are mainly concentrated in coastal East Asia and Europe, with China, the United Kingdom, and Germany accounting for most detected turbines. This pattern reflects differences in offshore wind resource development and policy support, as well as the influence of marine engineering conditions and grid connection feasibility. The bathymetric analysis provides additional evidence of these engineering constraints. Asian countries, represented by China and Vietnam, concentrate more OWTs in shallow nearshore waters, whereas major European offshore wind countries have more mature deployments in intermediate-depth zones. These differences suggest that turbine-level OWT inventories can support not only infrastructure monitoring but also comparative analyses of offshore wind development stages, siting preferences, and regional engineering strategies.

The proposed framework also has practical value for annual OWT inventory updating and multi-year offshore wind monitoring. Although the present implementation focuses on 2025, the annual compositing strategy is not tied to this specific year. By integrating valid multitemporal observations acquired throughout a year, annual compositing incorporates temporal information and reduces the sensitivity of the resulting SAR representation to short-term and seasonal variations among individual acquisitions. This improves the applicability of the representation across different years. Therefore, for other years, the same workflow can be applied by replacing the 2025 image collection with valid Sentinel-1 observations from the corresponding year. This design enables the framework to generate comparable annual OWT inventories and track the year-to-year expansion of offshore wind infrastructure, provided that sufficient valid SAR acquisitions are available.

Incorporating Sentinel-1 observations from multiple years may further improve OWT identification by providing repeated evidence of persistent offshore structures and reducing the influence of abnormal sea-state conditions or temporary acquisition gaps. However, multi-year compositing should be used cautiously because it may obscure the construction timing of newly installed turbines and reduce the temporal specificity of an annual inventory. Therefore, year-specific compositing is more appropriate for annual database updating, whereas multi-year observations can serve as auxiliary temporal consistency information to reduce uncertainty.

### 4.2. Methodological Advantages of CPEF Framework

The methodological advantage of CPEF lies in combining candidate-guided search with structure-aware refinement. In global-scale SAR imagery, OWTs occupy only a small proportion of the image area, whereas most pixels represent homogeneous sea surface background. Direct dense inference with a deep network [29,30,31,32] would therefore introduce substantial computational redundancy, forcing the model to process many information-poor regions [33]. In this framework, TM-CFAR serves primarily as an efficient candidate generator. It rapidly narrows the search space and transforms global OWT mapping from full-scene dense detection into candidate-level discrimination. This design enables the second-stage model to focus on target-relevant regions rather than extensive sea surface backgrounds, thereby improving the balance between large-area processing efficiency and fine discrimination accuracy.

**Figure 9:**
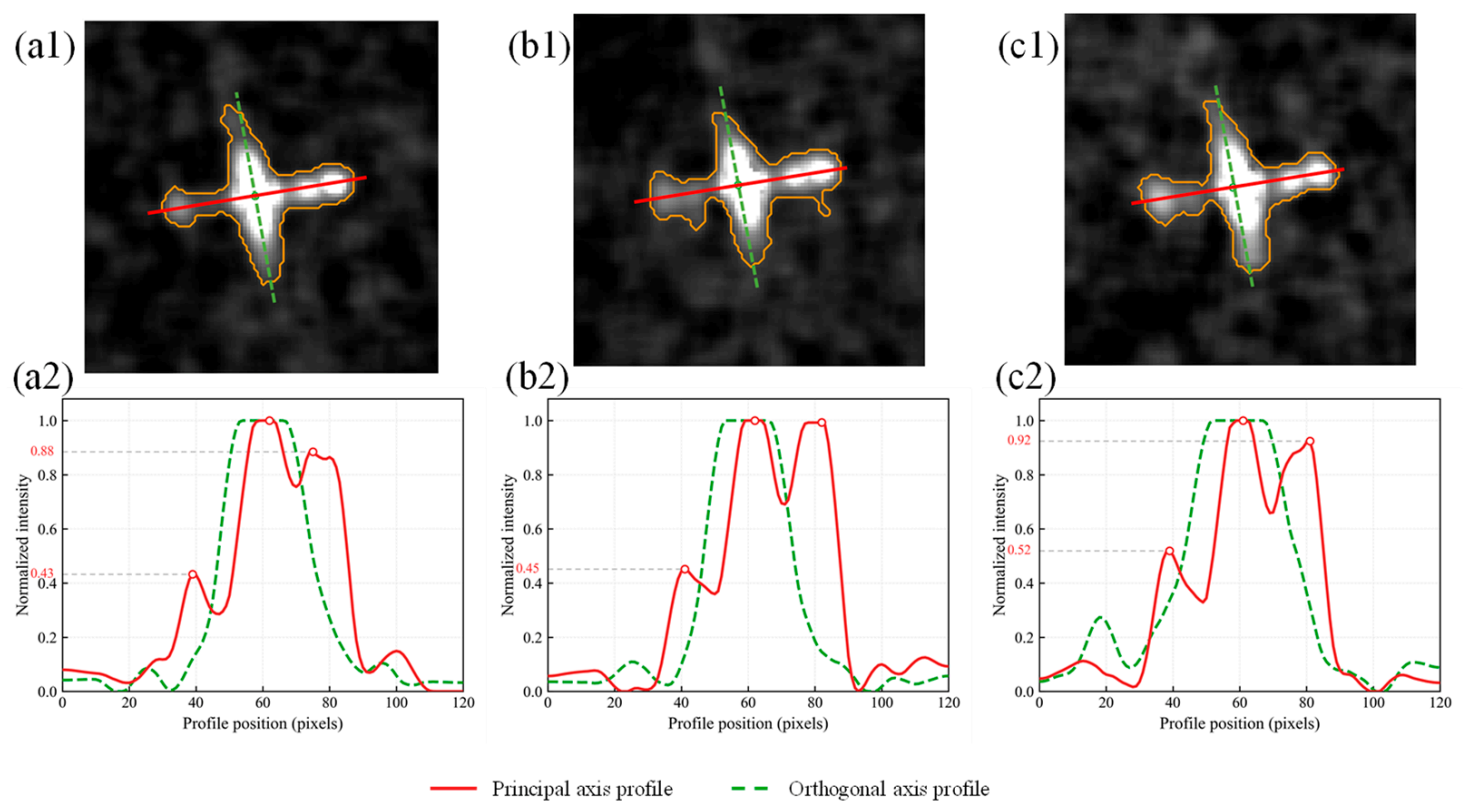
The PCA-aligned profile features of three representative OWT candidates with different local scattering patterns. (**a1**) shows the local SAR response, high-response contour, and PCA-derived axes of Candidate A, (**a2**) shows a broad central peak with asymmetric side peaks. (**b1**) shows the corresponding spatial features of Candidate B, (**b2**) exhibits two distinct peaks along the principal axis. (**c1**) shows the spatial features of Candidate C, (**c2**) presents an asymmetric principal-axis profile with multiple peaks. The red solid and green dashed curves represent the principal- and orthogonal-axis profiles, respectively.

More importantly, the main methodological contribution of CPEF is the introduction of PCA-aligned peak profile features into candidate refinement. Previous threshold-based SAR mapping methods efficiently exploit the strong backscatter of OWTs, but local intensity alone is insufficient to distinguish real turbines from turbine-like bright targets [21]. Although convolutional neural networks can learn texture, edge, and contextual features from local image patches [34], their decision basis is largely implicit and may become unstable when background conditions or target-like structures differ from those in the training data [35]. In contrast, the proposed PCA-aligned peak profile branch explicitly describes the structural organization of scattering peaks along the principal and orthogonal directions of each candidate. It therefore provides a physically interpretable structural prior that complements deep image features. As illustrated in Figure 9a1–c2, principal axis alignment makes the local peak structure difference between OWTs and turbine-like false targets more directly comparable in a unified reference frame. Compared with approaches that rely mainly on farm-scale array patterns [36], this candidate-level structural representation is more flexible because it does not depend on the overall layout regularity of an entire wind farm and can therefore better adapt to diverse coastal environments and wind farm configurations.

To further evaluate the contribution of the PCA-aligned peak profile features, we conducted an ablation experiment comparing an EfficientNet-B0 image-only model with the proposed fusion model. As shown in Table 1, incorporating the PCA-aligned peak profile features improved accuracy from 92.00% to 97.00%, precision from 90.38% to 96.08%, recall from 94.00% to 98.00%, and the F1-score from 92.16% to 97.03%, corresponding to increases of 5.00, 5.70, 4.00, and 4.87 percentage points, respectively. These improvements indicate that the PCA-aligned peak profile features improve both the correct identification of true OWTs and the suppression of non-turbine false targets. In particular, the increase in precision suggests that the structural features help reduce false positives caused by turbine-like strong scatterers, while the improvement in recall indicates that the method retains true turbines more completely. Overall, the ablation results confirm that the PCA-aligned peak profile branch provides effective structural information beyond image texture and improves the discriminative ability of the CPEF framework.

### 4.3. Limitations and Future Work

Despite its effectiveness, the proposed framework has several limitations. First, the current preprocessing strategy and TM-CFAR parameter settings were developed based on Sentinel-1 SAR imagery. Their transferability to other SAR sensors requires further validation. For example, high-resolution X-band observations from TerraSAR-X/TanDEM-X may enable a more detailed characterization of turbine-scale scattering structures [37]. ALOS-2 PALSAR-2 provides L-band SAR observations, but its scattering characteristics and acquisition strategy differ substantially from those of Sentinel-1 [38]. Therefore, applying CPEF to other SAR sensors may require the recalibration of the preprocessing strategy and TM-CFAR parameters. Future work could explore adaptive candidate generation and data-driven threshold learning to reduce manual parameter tuning and improve the framework’s adaptability to multi-source SAR imagery.

Second, the temporal robustness of the framework has not yet been fully evaluated. Although annual compositing can reduce short-term fluctuations by integrating all valid Sentinel-1 observations within a year, this study did not separately quantify detection performance across years, months, or seasons. In practice, sea state, wind conditions, wave state, vessel activity and acquisition density may vary among years or seasons, thereby changing the local contrast between turbines and the sea background in individual SAR images. Future work could apply the same workflow to additional annual Sentinel-1 collections, including data from 2024 and earlier years, to evaluate cross-year robustness. Monthly or seasonal validation experiments could also be conducted to further assess the sensitivity of OWT detection to temporal variations. In addition, time-series change detection methods such as LandTrendr [39] and Continuous Change Detection and Classification [40] could be integrated to estimate the construction timing and expansion. This would support the development of a temporally explicit global database of OWT dynamics.

Finally, this study focuses primarily on turbine-level planar location mapping and does not retrieve three-dimensional structural or engineering attributes, such as turbine height, rotor diameter, installed capacity, or operational status. Future research could combine SAR data with optical imagery, engineering databases, nighttime lights, or other auxiliary data to expand the attribute dimensions of the OWT inventory. Methods developed for building height or infrastructure parameter estimation [10,41,42] may also provide useful methodological references. Such extensions would increase the value of the dataset for offshore wind capacity estimation, environmental impact assessment, marine spatial planning, and the long-term monitoring of offshore wind development.

## 5. Conclusions

This study developed CPEF, a two-stage framework for large-scale OWT detection using Sentinel-1 SAR imagery. The framework first uses TM-CFAR to generate high-recall candidate regions from annual SAR composites and then refines them with an EfficientNet-B0-based classifier. By shifting the task from full-scene dense inference to candidate-level discrimination, CPEF reduces the redundant processing of homogeneous sea surface backgrounds while preserving sensitivity to turbine-like targets. The main methodological contribution of CPEF is the explicit incorporation of PCA-aligned peak profile features into the refinement stage. Rather than relying solely on the image texture learned implicitly by the CNN, the proposed model encodes the axial scattering organization of OWTs along the principal and orthogonal directions. It then fuses these structural descriptors with deep visual features. This design strengthens discrimination between true turbines and strongly scattering false targets, such as offshore substations and oil platforms, while enhancing the interpretability of the classification process.

Using the CPEF framework, we updated the global OWT spatial distribution dataset through 2025 and identified 16,270 turbines. This total exceeds the 15,606 turbines in the available DeepOWT dataset and fills detection gaps in regions including Jiangsu, Shanghai, and the Dogger Bank area of the North Sea. Accuracy assessment demonstrated strong performance in OWT detection. Overall accuracy (OA), user’s accuracy (UA), producer’s accuracy (PA), and the F1-score were 96.33%, 97.50%, 97.01%, and 97.25%, respectively, indicating high detection accuracy and stability. Further spatial analysis revealed the pronounced large-scale regional clustering of global OWTs, concentrated primarily in the nearshore waters of Europe and coastal East Asia, and successfully captured the regular array layouts characteristic of typical OWFs. Water depth analysis indicated that global OWTs overall tend to cluster in shallow, nearshore, and semi-enclosed waters, with development still concentrated in shallow and intermediate-depth zones across countries. Cross-country differences in water depth distribution are jointly shaped by engineering costs, foundation types, and the stages of industrial development. Moreover, the spatial tendency of global OWFs to agglomerate along the margins of semi-enclosed shallow seas reflects that current offshore wind development remains largely constrained by natural conditions, engineering feasibility, grid connection and maintenance requirements. Overall, the proposed CPEF framework provides an efficient, reproducible, and structurally interpretable approach for identifying OWTs in global large-scale SAR imagery. The updated global OWT spatial distribution dataset not only supports the updating of offshore wind databases but also offers a reliable data foundation for spatial pattern analysis, resource potential assessment, and future spatiotemporal evolution studies of global offshore wind energy.

## Figures and Tables

**Table 1:** Ablation analysis of PCA-aligned peak profile features.

Model	PCA-Aligned Peak Profile Features	Accuracy (%)	Precision (%)	Recall (%)	F1-Score (%)
EfficientNet alone	No	92.00	90.38	94.00	92.16
EfficientNet-B0 and peak profile	Yes	97.00	96.08	98.00	97.03

## Data Availability

The data were uploaded at https://doi.org/10.5281/zenodo.20572450 (accessed on 6 June 2026).

## Abbreviations

The following abbreviations are used in this manuscript:

CPEF	CFAR-Guided PCA-Aligned Peak Profile and EfficientNet Fusion
OWT	Offshore Wind Turbine
SAR	Synthetic Aperture Radar
EEZ	Exclusive Economic Zone
TM-CFAR	Trimmed Mean Constant False Alarm Rate
